# Contralateral Breast Cancer With Multiple Primary Neoplasms in a Patient With Neurofibromatosis Type 1: A Case Report and Review of the Literature

**DOI:** 10.7759/cureus.13738

**Published:** 2021-03-06

**Authors:** Shifaz M Veettil, Jawaid Younus, Edward Yu

**Affiliations:** 1 Oncology, Schulich School of Medicine & Dentistry, Western University, London, Ontario, CAN; 2 Medical Oncology, Schulich School of Medicine & Dentistry, Western University, London, Ontario, CAN; 3 Radiation Oncology, Schulich School of Medicine & Dentistry, Western University, London, Ontario, CAN

**Keywords:** oncology, radiation oncology, medical oncology, breast cancer, neurofibromatosis type 1

## Abstract

Neurofibromatosis type 1 (NF1) is an autosomal dominant neuroectodermal disorder associated with increased risk for several neural and non-neural malignancies. The link between NF1 and breast cancer has recently been established, with patients with NF1 being at higher risk for developing breast cancer, more likely to get breast cancer at a younger age, and more likely to have their breast cancer present with more adverse prognostic factors. Although rare, several cases of NF1 patients with contralateral breast cancer have been mentioned in the literature. We report the case of one such patient who developed contralateral breast cancer 40 years after her initial breast cancer diagnosis.

## Introduction

Neurofibromatosis type 1 (NF1) is an autosomal dominant neuroectodermal disorder caused by heterozygous loss-of-function mutations of the tumour suppressor gene *NF1* on chromosome 17q11.2. The prevalence of NF1 ranges from 1 in 2000 to 6000 individuals and is associated with an estimated eight- to 15-year decrease in life expectancy [1-2]. The disorder is classically characterized by café-au-lait spots, axillary freckling, and both dermal and plexiform neurofibromas. Patients with the condition are at four times greater risk for developing malignancy and are especially predisposed to tumours of the peripheral and central nervous systems [3]. In addition, NF1 has been associated with non-neural cancers such as pheochromocytoma, leukemia, and rhabdomyosarcoma. Recent reports have also linked NF1 with breast cancer, with several identifying the development of contralateral breast cancer in breast cancer patients with NF1. In this report, we examine the case of a patient with NF1 who developed contralateral breast cancer with multiple primary neoplasms four decades after her initial diagnosis of breast cancer.

## Case presentation

**Figure 1:**
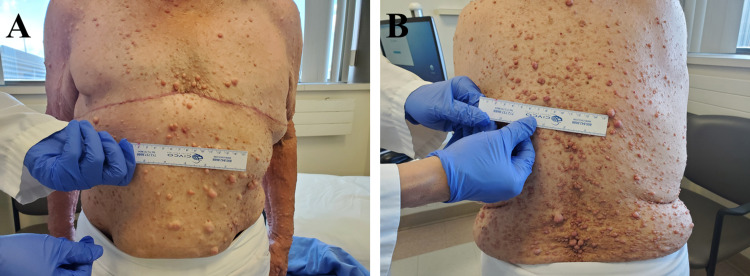
Adult presentation of neurofibromatosis type 1 (NF1) with anterior (A) and posterior (B) views showing significant neurofibromas on the chest, trunk, and upper limbs.

A 74-year-old woman was referred to our center after identification of multiple masses in her left breast on screening mammography and subsequent left mastectomy. She was diagnosed with NF1 during childhood and displayed neurofibromas on her face, chest, trunk, and upper limbs (Figure 1). The patient had previous right-sided breast cancer at 34 years of age with positive lymph node involvement, positive estrogen receptor (ER) status, and negative progesterone receptor (PR) status. She was subsequently treated with right mastectomy, adjuvant chemotherapy, and adjuvant tamoxifen at that time. She had her first menstrual period at 14 years of age and reached menopause at 50 years of age. No history of hormone replacement or oral contraceptives was reported. She denied smoking in the past and only occasionally drank alcohol. The only family history of breast cancer that was noted was in a cousin and a niece on her mother’s side, and there was no documented family history of NF1.

The patient underwent bilateral screening mammographs after experiencing a rupture of her already present right breast implant, which found three to four masses in her left breast, with the largest being 2.9 cm. Subsequent ultrasound-guided core needle biopsies were procured, which suggested multiple masses of distinct pathologies. The patient then underwent left mastectomy and sentinel lymph node biopsy. Further workup with chest and abdominal computed tomography (CT) as well as bone scan detected no distant metastasis. Pathology confirmed the diagnosis of left breast invasive ductal carcinoma with two separate foci, the larger being a 44 mm tumour at stage pT2, pN1a (grade III) and with positive ER and PR statuses, and the smaller tumour measuring 7 mm at stage pT1b, pN0 (grade II) with triple-negative status. Her case was reviewed at the weekly Breast Multidisciplinary Team Tumour Board meeting and it was proposed that she be initially started on adjuvant chemotherapy - three cycles of fluorouracil, epirubicin, and cyclophosphamide, followed by three cycles of docetaxel - and then receive loco-regional radiation therapy and hormonal therapy.

## Discussion

Most cases of NF1 are detected in childhood, and diagnosis can be established when two or more of the criteria set out by the National Institutes of Health are met (Table 1) [4]. These guidelines were determined to be of both high specificity and sensitivity in adults with NF1, and diagnosis can typically be made with just history and physical exam alone.

**Table 1:** Diagnostic criteria for neurofibromatosis type 1 (NF1).

Criteria	
1	Six or more café au lait macules >5 mm in greatest diameter in pre-pubertal individuals and >15 mm in greatest diameter in post-pubertal individuals
2	Two or more neurofibromas of any type or one plexiform neurofibroma
3	Freckling in the axillary or inguinal regions
4	Optic glioma
5	Two or more Lisch nodules (iris hamartomas)
6	A distinctive osseous lesion such as sphenoid dysplasia or tibial pseudarthrosis
7	A first-degree relative (parent, sibling, or offspring) with NF1 as defined by the above criteria

The genetic basis of NF1 has been localized to mutations in the *NF1* gene, and the disorder presents with full penetrance but with complex, multi-organ, and widely variable expression [5]. The *NF1* gene has been identified as a tumour suppressor gene due to its involvement in the Ras pathway, with *NF1* dysfunction leading to Ras overexpression - significant as Ras overexpression is responsible for as many as 60% of breast cancer cases [6-7]. The *NF1* gene is located on the long arm of chromosome 17, which also contains the *BRCA1* gene, and some have suggested possible concomitance of NF1 and hereditary breast cancer, though few cases of breast cancer in patients with both NF1 and *BRCA1* mutations have actually been reported [8-10]. *NF1*’s involvement in breast cancer was eventually confirmed through the Cancer Genome Atlas program [7].

Previously, although the association between NF1 and cancers of the nervous system was clearly defined, the link between the disorder and breast cancer was just hinted at with only case reports to rely on [11]. However, more recent studies have since established a strong clinical association between NF1 and breast cancer [12-13]. This risk of developing breast cancer was particularly elevated for younger patients - such as our patient for her initial breast cancer diagnosis - with 11.1-times increased risk in NF1 patients younger than 40 years of age [13]. Women with NF1 also experience at least a 3.5-fold elevated mortality from breast cancer as well as worse five-year survival compared to the general population [2]. In addition to an earlier age of typical onset, NF1 patients that develop breast cancer tend to be diagnosed at higher T stages compared to the general population [7]. These patients were also determined to more likely have additional adverse prognostic factors for breast cancer, such as ER negativity, PR negativity, and human epidermal growth factor receptor 2 (HER2) positivity [7,14]. The significant number of dermal neurofibromas characteristic of NF1 also exacerbates the difficulty of early identification of disease as they can mask breast lesions on examination and mammography, leading to delay in diagnosis and thus worse outcomes [15].

Although rare, just like with our patient, several cases of contralateral breast cancer have been identified in the literature (Table 2). It was previously known that mutations in other genes known to predispose to breast cancer, such as *BRCA1/2*, result in an increased risk of contralateral breast cancer [16]. However, it was not until recently that a similar pattern was established for alterations in *NF1*, with a cohort study finding a two-fold increased risk for women with NF1 compared to women without hereditary breast cancer-related mutations, though this risk was still half that of women with *BRCA1/2* mutations [17]. The same study found that more than a quarter of women with NF1 at two decades of survival from their initial breast cancer are likely to have developed contralateral breast cancer, leading the authors to recommend consideration of preventative contralateral mastectomy for NF1 patients with breast cancer. Our patient is notable as she developed contralateral breast cancer 40 years after her initial breast cancer.

**Table 2:** Cases of contralateral breast cancer in patients with neurofibromatosis type 1 (NF1). DCIS, ductal carcinoma in situ; IDC, invasive ductal carcinoma

Patient	Sex	Breast Cancer Histology (age at diagnosis)
1	Female	IDC, right breast (34 years); IDC, left breast (74 years); IDC, left breast (74 years)
2 (Dursun et al., 2017) [10]	Female	IDC, left breast (26 years); IDC, right breast (42 years)
3 (Sharif et al., 2007) [12]	Female	IDC (47.7 years); IDC (53.2 years)
4 (Sharif et al., 2007) [12]	Female	IDC (34.4 years); IDC (36.4 years)
5 (Wilson et al., 2004) [18]	Male	DCIS, left breast (18 years); DCIS, right breast (18 years)
6 (Takeuchi et al., 2011) [19]	Female	IDC, left breast (69 years); Lobular, right breast (76 years)
7 (Wang et al., 2012) [20]	Female	DCIS, left breast (40 years); DCIS, right breast (43 years); DCIS, left breast (44 years)
8 (Wang et al., 2012) [20]	Female	IDC, right breast (43 years); IDC, left breast (47 years)

## Conclusions

Neurofibromatosis type 1 (NF1) is an autosomal dominant neuroectodermal disorder associated with increased risk of neural and non-neural malignancies, including breast cancer. The link between NF1 and breast cancer is now well recognized, both biologically and clinically, with NF1 patients having greater risk, more adverse prognostic risk factors, and increased mortality for breast cancer. A few cases of NF1 patients with contralateral breast cancer have been identified in the literature, and our case highlights an NF1 patient who developed contralateral breast cancer nearly four decades after her initial breast cancer diagnosis. It has been suggested that NF1 patients may be at higher risk of developing contralateral breast cancer. Given that significant dermal neurofibromas can mask breast lesions, routine mammogram and physical examination for surveillance may not be sufficient. It is the authors' recommendation that a thorough discussion should be offered to NF1 patients on the risk of breast cancer development and the potential of contralateral breast cancer risk. Consideration of preventative contralateral mastectomy for NF1 patients at risk may be warranted.
